# Gait Indices for Characterization of Patients with Unilateral Cerebral Palsy

**DOI:** 10.3390/jcm9123888

**Published:** 2020-11-30

**Authors:** Stefanos Tsitlakidis, Martin Schwarze, Fabian Westhauser, Korbinian Heubisch, Axel Horsch, Sébastien Hagmann, Sebastian I. Wolf, Marco Götze

**Affiliations:** Clinic of Orthopedics and Trauma Surgery, Heidelberg University Hospital, Schlierbacher Landstrasse 200a, 69118 Heidelberg, Germany; stefanos.tsitlakidis@med.uni-heidelberg.de (S.T.); martin.schwarze@med.uni-heidelberg.de (M.S.); fabian.westhauser@med.uni-heidelberg.de (F.W.); korbinian.heubisch@med.uni-heidelberg.de (K.H.); axel.horsch@med.uni-heidelberg.de (A.H.); sebastien.hagmann@med.uni-heidelberg.de (S.H.); sebastian.wolf@med.uni-heidelberg.de (S.I.W.)

**Keywords:** unilateral cerebral palsy, 3D-instrumented gait analysis, gait index, classification, unclassified patterns

## Abstract

As cerebral palsy (CP) is a complex disorder, classification of gait pathologies is difficult. It is assumed that unclassified patients show less functional impairment and less gait deviation. The aim of this study was to assess the different subgroups and the unclassified patients with unilateral CP using different gait indices. The Gillette Gait Index (GGI), Gait Deviation Index (GDI), Gait Profile Score (GPS) and spatiotemporal parameters derived from instrumented 3D-Gait Analysis (IGA) were assessed. Subgroups were defined using morphological and functional classification systems. Regarding the different gait indices, a ranking of the different gait patterns is evident. Significant differences were found between GMFCS level I and II, Winters et al. (Winters, Gage, Hicks; WGH) type IV and type I and the WGH-unclassified. Concerning the spatiotemporal parameters significant differences were found between GMFCS level I and II concerning velocity. The unclassified patients showed mean values for the different gait indices that were comparable to those of established subgroups. Established gait patterns cause different degrees of gait deviation and functional impairment. The unclassified patients do not differ from established gait patterns but from the unimpaired gait. Further evaluation using 3D-IGA is necessary to identify the underlying gait pathologies of the unclassified patients.

## 1. Introduction

Cerebral Palsy (CP) is a complex and heterogeneous disorder, leading to a variety of secondary musculoskeletal symptoms and deformities [1,2,3,4]. Specifically, in unilateral CP, other than in diplegic or quadriplegic CP, gait patterns may even depend on walking speed and on the extent of asymmetry [2,5,6]. Different classification systems (morphological and functional) have been developed in the past, in order to simplify treatment recommendations and decision-making [7,8,9,10]. Many of these classification systems are depicting either the gait pathology or the functional impairment mainly on a qualitative level without taking the degree of the impairment and the actual quantitative deviation [compared to the unimpaired gait of typically developing individuals (TD)] into account [11]. Furthermore, many classification systems are lacking reliability [8]. Particularly, considering that clinical/observational classification is difficult, there is a considerable number of patients that remain unclassified [12,13,14]. 

As the classification system of Winters et al. (Winters, Gage, Hicks; WGH) is based on morphological aspects, a conclusion concerning the caused functional impairment is not provided [10]. However, an increasing involvement from type I to type IV is evident [10]. Unclassified patients according to the classification system of WGH were assumed to show the least and undetectable gait pathology [15,16].

In the past, using instrumented 3D-Gait Analysis (IGA), efforts have been made to establish automated joint motion pattern recognition in order to improve gait classification [8,17,18]. Moreover, different gait indices derived from IGA have been introduced in order to quantify gait and gait abnormalities in comparison to the unimpaired gait of the TD [19,20,21,22,23].

The Gillette Gait Index (GGI) was the first gait index to be introduced. Schutte et al. introduced the GGI in order to summarize the complex and enormous amounts of data derived from IGA and to represent a patient’s gait abnormality from the average normal profile as a single number and originally was developed for the assessment of patients with CP. The GGI includes kinematic as well as spatiotemporal parameters, such as cadence and walking speed e.g., that rather describe walking ability than just the deviation from the unimpaired gait of the TD [19,20,23,24]. The authors describe a mean GGI value for their healthy controls of 15.7 [20]. Higher values represent a deviation from the unimpaired gait of the TD [20]. Furthermore, Romei et al. found a strong correlation between GGI and Gross Motor Function Measure (GMFM) [25]. The Gait Deviation Index (GDI) was described by Schwartz et al. in order to quantify the degree of gait abnormality compared to the unimpaired gait of the TD and is mostly used compared to the other gait indices [21]. A value of 100 represents a normal and unimpaired gait, whereas every 10 points less represent one standard deviation and therefore a significant impairment [21,26]. Furthermore, the GDI shows an excellent correlation with the different Gross Motor Function Classification System (GMFCS) levels and is therefore not only capable to depict the morphological deviation, but the functional impairment as well [26,27,28]. Additionally, the GDI is capable to represent the walking capacity and activity, as strong correlation between these functional parameters and the GDI were found [29,30]. The Gait Profile Score (GPS) was developed including the same kinematic features as the GDI [19]. However, the GPS differs mathematically from the GDI and represents a raw score rather than a logarithmic scaled value [19,21]. A patient with data or a value that is twice as different from that of another person shows twice the GPS [19]. The GDI and the GPS were developed for gait assessment of patients in general and not exclusively for CP patients [19,21,23,31,32]. Furthermore, the GGI and the GDI were suggested as an adequate parameter for follow-up evaluation of gait and correction procedures [33,34,35].

The objective of this current study was the collection, assessment and correlation of the different gait indices in a cohort of patients with unilateral CP in order to compare the different subgroups according to the GMFCS and the WGH classification system against each other and to characterize the unclassified patients.

## 2. Materials and Methods

This study was conducted as a register study after approval by the local ethics committee (S-198/2019).

Only patients with unilateral CP and a GMFCS level I-II were included retrospectively [11]. Instrumented 3D-gait analysis (IGA) was performed from 2006 to 2017 using a 120-Hz 9-camera system (Vicon, Oxford Metrics, Oxford, UK) and two piezoelectric force plates (Kistler, Winterthur, Switzerland). Reflective markers were applied to bony landmarks according to the protocol of Kadaba et al. [36]. All participants were asked to walk barefoot at a self-selected speed along a seven-meter walkway. The data was extracted from our motion laboratory data base.

Finally, 89 patients (40 female, 49 male) matched the inclusion criteria (unilateral CP, no previous surgery of the lower limbs, no Botulinumtoxin–A injections within the last six months, GMFCS I–II) with a mean age at the time of IGA of 15.3 ± 9.6 years were included in this study [11]. This cohort has been established previously [11].

All participants were classified according to the GMFCS [7] as well as according to the classification system of WGH [10] separately. Additionally, in order to define further subgroups, the patients of the different WGH types (type I–IV) were discriminated using the GMFCS within the WGH types in a second step (Table 1).

Subsequently, the Gait Deviation Index (GDI) [21], Gillette Gait Index (GGI) [20] and Gait Profile Score (GPS) [19] were calculated/derived from IGA for the impaired limbs. The gait indices were compared against each other in order to investigate for potential quantitative differences and to evaluate the caused functional impairment and the degree of gait deviation using the strong correlation of the gait indices with functional classification systems. Furthermore, the gait indices were evaluated for further characterization of patients that did not meet classification criteria of the WGH classification system.

Additionally, spatiotemporal parameters such as stance phase duration, velocity and cadence were assessed, as they rather represent walking ability. To avoid interindividual differences, velocity and cadence were normalized respect to leg length according to Hof et al. [37].

### 2.1. Statistical Analysis

Data was structured using Microsoft Excel (Microsoft, Redmond, WA, USA) and analyzed using SPSS Version 25.0 (IBM, Chicago, IL, USA). For descriptive statistics, the mean and the standard deviation (SD) were calculated.

#### 2.1.1. Correlation Analysis

The Pearson product moment correlation coefficient (PPMC) was calculated for the correlation analysis of the different gait indices of the total cohort (GDI vs. GPS, GDI vs. GGI, GPS vs. GGI).

#### 2.1.2. Comparative Statistics of GMFCS Levels

Comparative statistics for the comparison of GMFCS level I and level II (with respect to the gait indices and spatiotemporal parameters) was performed using the two-tailed student’s T-test. The level of significance was set at *p* < 0.05.

#### 2.1.3. Comparative Statistics of WGH Types

For the comparison of the different WGH types (with respect to the gait indices and spatiotemporal parameters), ANOVA test was performed, followed by Bonferronis’s post hoc test. The level of significance was set at *p* < 0.05.

#### 2.1.4. Comparative Statistics of GMFCS Levels within the Different WGH Types

The two-tailed student’s *t*-test was used as well in order to compare GMFCS level I and II within the different WGH types (WGH type I_GMFCS level I vs. WGH type I_GMFCS level II, WGH type II_GMFCS level I vs. WGH type II_GMFCS level II, etc.). The level of significance was set at *p* < 0.05.

## 3. Results

Table 1 displays the mean values of the different gait indices and of the different spatiotemporal parameters for every subgroup. Due to the small sample size of *n* = 2 the WGH type III subgroup was excluded from further comparative statistics (Table 2, Table 3 and Table 4).

With regard to the different gait indices, GMFCS level I and WGH type I subgroups showed the lowest GGI values and the highest GDI values (Table 1). As the GPS and the GDI include the same kinematic features, the relation of mean values for the different subgroups was similar. The unclassified patients showed mean values for the different gait indices that were comparable to those of the WGH type I, thus still showing considerable deviation from the unimpaired gait of the TD (Table 1).

The differences concerning the spatiotemporal parameters appeared to be less pronounced between the different subgroups (Table 1).

### 3.1. Correlation Analysis

While the correlation between the GDI and the GPS was excellent (r = −0.847), moderate correlation coefficients between the GDI and the GGI (r = −0.308) and between the GPS and the GGI (r = 0.364) were evident.

### 3.2. Comparative Statistics of GMFCS Levels

Further comparative statistics revealed that differences between GMFCS level I and II were statistically significant for all gait indices (Table 2) and for velocity (Table 3).

### 3.3. Comparative Statistics of WGH Types

The results for WGH type IV showed a statistically significant greater deviation compared to WGH type I and WGH-unclassified for the GDI and the GPS and additionally compared to WGH type II for the GGI (Table 2). There were no statistically significant differences concerning spatiotemporal parameters between WGH subtypes (Table 3).

### 3.4. Comparative Statistics of GMFCS Levels within the Different WGH Types

Significant differences between the GMFCS levels within the different WGH subtypes were found only for WGH type II concerning the GPS (Table 4). Interestingly, differences between the GMFCS levels within the unclassified patients were emphasized and were found for the GDI, the GPS and stance phase duration (Table 4).

## 4. Discussion

As CP is complex and CP patients are very heterogeneous, a variety of gait patterns with different degrees of severity and functional impairment result [1,38]. Current classification systems often depict either the gait pathology morphologically or the functional impairment mainly on a qualitative level [7,8,9,10]. In the past, different gait indices have been developed for quantification of the abnormal gait in comparison to the unimpaired gait of the TD [19,20,21].

The objective of this current study was the collection, assessment and correlation of the different gait indices for different subgroups of patients with unilateral CP. Moreover, various spatiotemporal parameters were assessed. The intent of our study was to put the different gait patterns and subgroups in relation to one another quantitatively and to characterize the unclassified patients.

Concerning the correlation analysis, excellent results between the GDI and the GPS were found. This can be explained by the fact that the GDI and the GPS are calculated from the same joint kinematic variables and are closely linked to one another [19,21,23,39]. As expected, moderate and quite similar correlation coefficients between the GGI and the GDI and between the GGI and the GPS were found. In contrast to the GDI and GPS, the GGI includes additional spatiotemporal parameters which explains the less pronounced correlation between GGI and GDI as well as GPS [20,23].

With respect to the different gait indices, a ranking of the different gait patterns is evident (Table 1). The obtained absolute values indicate that WGH type I, the WGH-unclassified, WGH type III and WGH type II showed comparable and statistically significant less deviation compared to WGH type IV and seem to be less impairing functionally (Table 1 and Table 2). Schutte et al. report in their first description of the GGI comparable mean values for WGH subtypes I and II during their first application of the GGI [20]. WGH types III and IV showed mean GGI values that were up to twice as high compared to our results [20]. The highest mean GDI value still was two SD below the unimpaired gait of the TD. The postulated increasing involvement [10,21,33] and therefore increasing gait deviation from type I to type IV was evident from the obtained results, though not statistically significant between type I and type II (Table 1).

Concerning the GMFCS as a functional classification system, level II patients showed significantly more deviation for all different gait indices compared to GMFCS level I patients (Table 1 and Table 2). GMFCS level I patients showed an average GDI value that is two SD below the unimpaired gait of the TD, whereas GMFCS level II patients showed a mean GDI value that is even one SD lower than level I (Table 1). This, vice versa indicates that the GDI is capable of depicting functional impairment. Our results are supported by previous findings [19,26,40]. Malt et al. and Maanum et al. report identical mean GDI values for GMFCS levels I and II [26,40]. Additionally, though evaluating patients with bilateral CP, Ito et al. described the same mean values for the different GMFCS levels as found during our investigation [41]. It seems, that the GDI might reflect GMFCS levels independently from the underlying neurological disorder. Furthermore, our observed mean GPS values for the different GMFCS levels were comparable to those observed by Baker et al. [19].

With respect to the different spatiotemporal parameters, significant differences were found only between GMFCS level I and GMFCS level II concerning velocity and stance phase duration between GMFCS level I and GMFCS level II within the unclassified patients (Table 1, Table 2 and Table 3). Absolute differences were clinically negligible. Comparable findings were reported by Zhou et al. and Ounpuu et al. [42,43]. In their study introducing a deviation index based on spatiotemporal parameters, Zhou et al. described a similar range for mean velocity and mean cadence [43]. However, the included patients with CP were grouped by age and not by GMFCS levels or morphological subtype [43]. In contrast, Ounpuu et al. grouped their patients by GMFCS levels [42]. Cadence as well as velocity for levels I and II matched our findings [42].

Various authors stated that patients with unilateral CP that do not meet the classification criteria represent a subgroup that is less involved and show non-detectable gait disorders [15,16]. Our results indicate that the unclassified patients do not represent a subgroup that shows no or negligible gait abnormalities, as they show comparable values for the different gait indices as other established gait patterns. Therefore, it seems that those patients have considerable gait abnormalities, which are not considered by established classification systems. Their gait patterns do differ considerably from the unimpaired gait of the TD. This indicates that the unclassified patients represent a heterogeneous cohort of patients with different degrees of gait deviation and impairment. This heterogeneity becomes clear regarding the GMFCS level-specific gait indices within the unclassified patients in particular. Concerning the GDI and the GPS, highly significant differences were evident (Table 1 and Table 4).

Patients with unilateral CP often show subtle gait disorders that are difficult to be detected [12,13,14]. Apart from that, as the often used and established classification systems mainly consider sagittal plane kinematics, detection of all possible gait deviations is restricted [3,8]. However, assigning patients into specific and predominant gait patterns and subgroups may be a significant simplification, as in reality gait deviations are far more complex and divers [2,8,44]. However, the used gait indices seem to detect gait deviations more accurate than the WGH classification system, even though they as well consider just a selection of parameters derived from IGA. Conclusions regarding the underlying morphological gait disorder still cannot be made.

Furthermore, Sagawa et al. described a relationship of clinical parameters—such as the strength of the hip extensor, the level of spasticity and the strength of the tibialis posterior—and severe gait deviation indicated by low GDI values [45]. However, these clinical parameters are not considered by the established/used classification systems for unilateral CP.

The used gait indices have not been without criticism. They are compromised by including just an arbitrary selection of parameters derived from IGA disregarding kinetic and electromyographic data [19,20,21]. Furthermore, the gait indices are sensitive to lab-specific control data leading to large differences of mean values and variance and might miss relevant gait changes [21,39].

Main limitation of this current study is the fact that the patients were included retrospectively and the absence of a control group of TD. However, IGA represents the “gold standard” in the evaluation of motion/gait disorders and is highly standardized, which allows a reliable and proper comparison evaluation. Nevertheless, possible methodological differences among laboratories might result in different gait measures and scores between the different laboratories compromising inter-study comparability. Moreover, on account of the small sample size of patients allocated to WGH type III, no conclusions can be drawn concerning this specific subgroup.

## 5. Conclusions

In conclusion, the different evaluated gait indices are useful for a quick general and overall characterization and quantification of gait abnormalities in patients with unilateral CP. Established gait patterns cause different and graduated degrees of gait deviation and functional impairment. Contrary to previous assumptions, the unclassified patients do not differ from the established gait patterns but from the unimpaired gait of the TD. Further evaluation using IGA is necessary in order to identify the underlying gait pathologies of the unclassified patients, e.g., specific and detailed evaluation of the gait patterns of the unimpaired limb and possible extrarticular malrotations.

## Figures and Tables

**Table 1:** Descriptive statistics. Subgroup-specific mean values of the different gait indices and mean spatiotemporal parameters.

Classification System	n	GGI (No Units)	GDI (No Units)	GPS (°)	Stance Phase Duration (%GC)	Norm. Velocity (m/s)	Norm. Cadence (steps/min)
**GMFCS**							
I	63	130.2 ± 116.0	80.2 ± 10.6	8.9 ± 3.0	58.7 ± 2.5	1.0 ± 0.2	108.5 ± 12.1
II	26	325.6 ± 438.5	70.2 ± 14.7	12.5 ± 4.2	59.4 ± 3.1	0.8 ± 0.2	104.2 ± 8.2
							
**WGH**							
**type I**	32	100.3 ± 67.8	81.6 ± 9.3	8.3 ± 2.3	59.4 ± 2.2	1.0 ± 0.2	107.2 ± 8.8
GMFCS I	26	91.2 ± 55.6	80.8 ± 8.2	8.2 ± 2.1	59.4 ± 2.1	1.0 ± 0.2	107.8 ± 9.2
GMFCS II	6	139.9 ± 103.6	84.8 ± 13.5	8.4 ± 3.2	59.4 ± 3.0	0.9 ± 0.2	104.6 ± 7.0
**type II**	19	157.0 ± 94.5	77.4 ± 11.7	10.2 ± 3.4	57.8 ± 2.3	0.9 ± 0.2	104.9 ± 10.0
GMFCS I	14	143.4 ± 87.1	80.4 ± 10.4	9.1 ± 2.6	58.0 ± 2.6	0.9 ± 0.2	105.6 ± 9.6
GMFCS II	5	195.0 ± 114.3	69.2 ± 12.4	13.4 ± 3.5	57.3 ± 1.4	0.8 ± 0.2	103.0 ± 12.1
**type III**	2	159.2 ± 149.7	80.0 ± 11.3	8.6 ± 2.7	59.3 ± 1.0	0.8 ± 0.1	103.6 ± 3.5
GMFCS I	1	53.4	88.0	6.7	60.1	0.89	101.2
GMFCS II	1	265.0	72.0	10.5	58.6	0.74	106.1
**type IV**	21	409.3 ± 474.2	68.1 ± 12.3	13.2 ± 4.2	59.3 ± 3.5	0.9 ± 0.2	107.3 ± 17.0
GMFCS I	10	297.7 ± 172.5	70.3 ± 12.3	12.2 ± 4.2	58.9 ± 4.1	0.9 ± 0.1	111.6 ± 22.8
GMFCS II	11	510.7 ± 631.8	66.1 ± 12.5	14.1 ± 4.0	59.7 ± 3.0	0.8 ± 0.2	103.3 ± 8.8
**unclassified**	15	104.2 ± 93.2	80.4 ± 15.6	8.8 ± 3.5	58.9 ± 3.0	1.0 ± 0.3	110.9 ± 12.8
GMFCS I	12	66.3 ± 34.3	86.2 ± 9.9	7.5 ± 1.7	57.9 ± 1.6	1.1± 0.2	111.5 ± 14.3
GMFCS II	3	255.9 ± 105.9	57.3 ± 12.9	14.1 ± 4.3	62.6 ± 4.6	0.8 ± 0.2	108.2 ± 3.1
**total**	89	187.3 ± 268.5	77.3 ± 12.7	9.9 ± 3.9	58.9 ± 2.7	0.9 ± 0.2	107.3 ± 12.0

GMFCS: Gross Motor Function Classification System. GGI: Gillette Gait Index. GDI: Gait Deviation Index. GPS: Gait Profile Score.

**Table 2:** Comparative statistics of the different gait indices between GMFCS levels and between WGH types. WGH: Winters, Gage, Hicks.

Classification System	*p*-Values GDI				*p*-Values GGI				*p*-Values GPS			
GMFCS (*t*-test)	I		II		I		II		I		II	
I	-		0.003		-		0.034		-		<0.001	
WGH (ANOVA/Bonferroni)	type I	type II	type IV	unclass.	type I	type II	type IV	unclass.	type I	type II	type IV	unclass.
type I	-	1.000	0.001	1.000	-	1.000	<0.001	1.000	-	0.498	<0.001	1.000
type II	/	-	0.145	1.000	/	-	0.015	1.000	/	-	0.063	1.000
type IV	/	/	-	0.028	/	/	-	0.004	/	/	-	0.001

/redundant value.

**Table 3:** Comparative statistics of the different spatiotemporal parameters between GMFCS levels and between WGH types.

Classification System	*p*-Values Stance Phase Duration				*p*-Values norm. Velocity				*p*-Values norm. Cadence			
GMFCS (*t*-test)	I		II		I		II		I		II	
I	-		0.259		-		<0.006		-		0.124	
WGH (ANOVA/Bonferroni)	type I	type II	type IV	unclass.	type I	type II	type IV	unclass.	type I	type II	type IV	unclass.
type I	-	0.510	1.000	1.000	-	1.000	0.346	1.000	-	1.000	1.000	1.000
type II	/	-	0.875	1.000	/	-	1.000	0.659	/	-	1.000	1.000
type IV	/	/	-	1.000	/	/	-	0.090	/	/	-	1.000

/redundant value.

**Table 4:** Comparative statistics between GMFCS levels for each WGH subtype. Each WGH subtype was further subgrouped according to GMFCS. Comparisons (using *t*-test) were done for all gait indices and all spatiotemporal parameters for WGH I_GMFCS I vs. WGH I_GMFCS II etc.

Classification System	*p*-Values GDI	*p*-Values GGI	*p*-Values GPS	*p*-Values Stance Phase Duration	*p*-Values Velocity	*p*-Values Cadence
	WGH Subtype_ GMFCS II	WGH Subtype_ GMFCS II	WGH Subtype_ GMFCS II	WGH Subtype_ GMFCS II	WGH Subtype_ GMFCS II	WGH Subtype_ GMFCS II
WGH I_GMFCS I	0.349	0.310	0.842	0.983	0.469	0.422
WGH II_GMFCS I	0.07	0.308	0.009	0.550	0.401	0.631
WGH IV_GMFCS I	0.446	0.316	0.304	0.608	0.431	0.279
unclass._GMFCS I	0.001	0.086	0.001	0.009	0.093	0.704
